# Primary Tumor Site Specificity is Preserved in Patient-Derived Tumor Xenograft Models

**DOI:** 10.3389/fgene.2019.00738

**Published:** 2019-08-13

**Authors:** Lei Chen, Xiaoyong Pan, Yu-Hang Zhang, Xiaohua Hu, KaiYan Feng, Tao Huang, Yu-Dong Cai

**Affiliations:** ^1^Shanghai Institute of Nutrition and Health, Shanghai Institutes for Biological Sciences, Chinese Academy of Sciences, Shanghai, China; ^2^College of Information Engineering, Shanghai Maritime University, Shanghai, China; ^3^Shanghai Key Laboratory of PMMP, East China Normal University, Shanghai, China; ^4^Department of Medical Informatics, Erasmus Medical Center, Rotterdam, Netherlands; ^5^Department of Biostatistics and Computational Biology, School of Life Sciences, Fudan University, Shanghai, China; ^6^Department of Computer Science, Guangdong AIB Polytechnic, Guangzhou, China; ^7^School of Life Sciences, Shanghai University, Shanghai, China

**Keywords:** Patient-derived tumor xenograft, gene expression profile, Monte Carlo feature selection, support vector machine, rule learning algorithm

## Abstract

Patient-derived tumor xenograft (PDX) mouse models are widely used for drug screening. The underlying assumption is that PDX tissue is very similar with the original patient tissue, and it has the same response to the drug treatment. To investigate whether the primary tumor site information is well preserved in PDX, we analyzed the gene expression profiles of PDX mouse models originated from different tissues, including breast, kidney, large intestine, lung, ovary, pancreas, skin, and soft tissues. The popular Monte Carlo feature selection method was employed to analyze the expression profile, yielding a feature list. From this list, incremental feature selection and support vector machine (SVM) were adopted to extract distinctively expressed genes in PDXs from different primary tumor sites and build an optimal SVM classifier. In addition, we also set up a group of quantitative rules to identify primary tumor sites. A total of 755 genes were extracted by the feature selection procedures, on which the SVM classifier can provide a high performance with MCC 0.986 on classifying primary tumor sites originated from different tissues. Furthermore, we obtained 16 classification rules, which gave a lower accuracy but clear classification procedures. Such results validated that the primary tumor site specificity was well preserved in PDX as the PDXs from different primary tumor sites were still very different and these PDX differences were similar with the differences observed in patients with tumor. For example, *VIM* and *ABHD17C* were highly expressed in the PDX from breast tissue and also highly expressed in breast cancer patients.

## Introduction

Patient-derived tumor xenograft (PDX) mouse models, developed by implanting patients’ *in vivo* tumor tissues into immune-deficient mice (Harris et al., 2016), are widely used in tumor biology and drug screening. Compared with cancer cell lines, PDX mouse models can maintain the original tumor development conditions immensely with appropriate tumor microenvironment that mimics similar regulatory factors, which are identified in the primary tumor site *in vivo* (Coats et al., 2017). Furthermore, with the development of humanized-xenograft models, PDX-humanized mouse models compensate for one of the prominent shortcomings of traditional PDX mouse models—the absence of immune regulation and selection—thereby accomplishing the accurate simulation on tumorigenesis *in vivo* (Jung et al., 2018).

As the PDX mouse model has more advantages in the oncology research field compared with traditional routines, various typical PDX mouse models have been successfully set up with their respective tumor tissues. Early in 2011, *Nature Medicine* published a systematic analysis (DeRose et al., 2011) on the pathological and biological characteristics of tumor tissues implanted into an immune-deficient mouse model as PDX. Such study confirmed that the PDX mouse model can basically reflect the same pathological processes during the initiation and progression of breast cancer, validating the significance of such model in the field of tumor research. Furthermore, PDX mouse models have been applied to various tumor subtypes, including colorectal cancer, pancreatic cancer, and pediatric cancer (Scott et al., 2017). Studies on such tumor subtypes have also confirmed that tumor tissues developed in a PDX mouse model have quite similar pathological and biological characteristics with tumor tissues in situ, though without immune selective pressure. Overall, PDX mouse models have been accepted as one of the most significant methods for tumor research.

In the field of oncology research, wide attention has been paid to gene expression characterizations. Different tumors have different expression pattern of functional tumor-associated genes as tumor-specific expression profile. Given the distinctive microenvironment and environmental selection pressure of human bodies and immune-deficient mice, the expression profile of a PDX mouse model has been confirmed to be different from the expression spectrum of tumor *in situ* (Ben-David et al., 2017). As mentioned above, different tumor subtypes have different tumor-specific expression profiles *in vivo*. However, after the selection and passaging in the mouse microenvironment, it is quite reasonable to speculate that tumor tissues of different subtypes may be differentially selected and lose/gain various differentially expressed genes (DEGs), thus generating a novel tumor subtype-specific expression profile (Ben-David et al., 2017). Although various studies have attempted to identify tumor subtype-specific biomarkers based on the expression profile of tumor tissues in PDX mouse models for years, no direct evidence or studies have revealed whether tumor tissues from different primary tumor subtypes can maintain tumor-specific DEGs during the passaging of PDX mouse models. Moreover, it is not clear whether such identified tumor-specific DEGs are all derived from the primary tumor tissues or from murine microenvironment selection.

To solve the problem, the most convenient way is to explore whether DEGs identified in PDX tumor tissues can still distinguish different tumor subtypes as potential biomarkers. Herein, we selected eight tumor subtypes originating from different tissues, including breast, kidney, large intestine, lung, ovary, pancreas, skin, and soft tissues, for the identification of DEGs in the PDX mouse model based on a study (Gao et al., 2015) on PDX tumor expression profile. Several advanced computational methods were used in this study, including the Monte Carlo feature selection (MCFS) (Draminski et al., 2008), incremental feature selection (IFS) (Liu and Setiono, 1998), and support vector machine (SVM) (Cortes and Vapnik, 1995). As a result, a group of highly related genes was identified, which may be distinctively expressed in different tumor subtypes as PDX tumor tissue. Furthermore, several quantitative rules were set up for the identification of different xenograft tumor subtypes by a specific set of functional distinctive genes. The results reported in this study further validated that PDX mouse models may be a relatively effective and practical mouse model in the field of tumor studies and may be favorable to be applied to indicate DEGs from primary tumor tissues between different tumor subtypes.

## Materials and Methods

### Dataset

We downloaded the expression data of 20,502 genes in eight PDX tumor tissues: (1) kidney, (2) skin, (3) ovary, (4) soft tissue, (5) breast, (6) pancreas, (7) lung, and (8) large intestine. The number of samples in each tissue is shown in **Table 1**. A total of 594 samples were considered in this study. The high-throughput screening data using PDX were obtained from the Gene Expression Omnibus (GEO) with accession number GSE78806 (Gao et al., 2015). To investigate whether the primary site of tumor has great influences on PDX, we compared the gene expression profiles of PDX from different primary sites.

**Table 1:** Number of samples for each of the eight tissues.

Tissue	Number of samples
Breast	79
Kidney	41
Large intestine	121
Lung	99
Ovary	52
Pancreas	94
Skin	46
Soft tissue	62
Total	594

### Feature Selection

Many genes are specifically expressed in the tissues; that is, some genes are closely related to certain tissues. To identify highly related genes for different tissues, we first used the MCFS (Draminski et al., 2008) method to analyze the expression data of 20,502 genes, obtaining a feature list and several classification rules. Then, the two-stage IFS (Liu and Setiono, 1998) method was applied to yield optimum features (genes), wherein the SVM (Cortes and Vapnik, 1995) exhibited a strong discriminative power for samples from different tissues.

#### Monte Carlo Feature Selection

MCFS (Draminski et al., 2008) is a type of feature selection method. As mentioned in the section *Dataset*, 594 samples were investigated in this study, and each sample was represented by 20,502 features. Thus, the dataset we studied is a high-dimensional dataset. The MCFS method is ideal in dealing with this type of dataset (Draminski et al., 2008). To date, this method has been applied to deal with several biological problems (Cai et al., 2018; Chen et al., 2018a; Chen et al., 2018c; Pan et al., 2018). In this study, it was also adopted to analyze all features and rank them for supervised classifiers.

MCFS constructs decision tree classifiers for many bootstrap sets that are randomly selected from the original sample set, and each tree is grown from a randomly selected feature subset with *m* features of original *M* features, where *m* is much less than *M*. During the process, *p* decision trees are generated on a training set randomly selected from a bootstrapping dataset and a feature subset. The above process is repeated *t* times to obtain *t* feature subsets. In total, *p* × *t* decision trees can be constructed.

The relative importance (RI) indicates the importance of each feature, which mainly considers the number of times that the feature is involved in growing the *p* × *t* decision trees. The RI score of a feature *g* can be calculated using the following formula:

(1) $$RI(g)=\sum_{\tau =1}^{pt}{\left(wAcc\right)}^{u}IG(n_{g}(\tau )){\left(\frac{no.in\;n_{g}(\tau )}{no.in\;\tau }\right)}^{v},$$

where *wAcc* is the weighted accuracy across all classes, *n* *_g_*(τ) indicates a node using feature *g* in decision tree τ, *IG*(*n* *_g_*(ᖟτ)) is the information gain of *n* *_g_*(ᖟτ), *no*.*in* τ is the number of training samples in τ, and *no*.*in* *n* *_g_*(ᖟτ) is the number of samples in node *n* *_g_*(τ). *u* and *v* are two weighting factors, and we used their default setting of *u* = *v* = 1.

A feature assigning a high MI value means that it is quite important. To extract most important features, the MCFS method adopts a permutation test on class labels. In detail, in a round of permutation test, a permutation of class labels is assigned to samples and the MCFS method is executed on the dataset with new labels, producing a maximal RI value. After several rounds, many maximal RI values are generated. The threshold, indicating high significance level of features, is determined by the one-sided Student’s *t* test. Features receiving the RI value larger than such threshold are selected and termed as informative features. These features are deemed to be essential for the investigated dataset. For a detailed description, please refer to Dramiński et al. (2011).

The informative features are extracted according to the essential properties of the dataset. However, for a given classifier, these features are not always optimal. Thus, we further ranked all features in a list according to their MI values in a way that features with high MI values receive high ranks in the list, whereas those with low MI values are placed at the bottom of the list. Here, we formulated the obtained feature list yielded by MCFS method as

(2) $$F=[f_{1},f_{2},\ldots ,f_{N}],$$

where *N* is the total number of features (*N* = 20,502 in this study). This list was used in the IFS method to select optimal features for a given classifier.

In this study, the program of the MCFS method was retrieved from http://www.ipipan.eu/staff/m.draminski/mcfs.html.

#### Rule Learning

Aside from analyzing features and ranking them in a list, the program of the MCFS method also integrates a rough set-based rule learning procedure. Based on informative features, the Johnson reducer algorithm (Ohrn, 1999) was used to select some important features that can give competitive classification performance compared with all informative features. After that, Repeated Incremental Pruning to Produce Error Reduction (RIPPER) algorithm (Cohen, 1995) produced the rules with the above-selected features. Each of these rules describes a relation between conditions (the left-hand side of the rule) and the outcome (the right-hand side). For example, a rule can be presented as an IF–THEN relationship based on expression values: IF Gene1 ≥ 6.4 AND Gene2 ≥ 4.8 THEN subtype = “kidney.” Following these rules, all samples can be easily classified. In addition, compared with black-box machine learning methods, the classification rules can provide a clearer classification procedure and help in understanding the expression differences among different tissues.

#### Incremental Feature Selection

The MCFS method only analyzes the importance of each feature and ranks them in a feature list. For a classification problem, it is necessary to extract some optimal features to comprise the feature subspace. Meanwhile, different classifiers require different optimal features. In view of this, the IFS (Liu and Setiono, 1998) method was employed in this study. The IFS method always integrates a supervised classifier to screen optimal features for accurately classifying samples from different groups. In the original IFS method, it first constructs a series of feature subsets according to a feature list in a way that the latter subset is produced by adding one feature to the former one. Then, for each feature subset, the supervised classifier is executed on the dataset, in which samples are represented by features in the subset. Finally, the feature subset yielding the best performance is selected as the optimal feature set. However, this procedure is time-consuming, especially when the number of features is quite large. Accordingly, we adopted a two-stage IFS method to approximately complete the procedure of finding optimal feature set in this study, which are described below.

In the first stage, several feature subsets with a large step (e.g., 10) were constructed. In detail, we constructed the feature subsets, denoted as $F_{1}^{1},F_{2}^{1},\ldots ,F_{m}^{1}$ where *m* = [*N*/10] and $F_{i}^{1}=\{f_{1},f_{2},\ldots ,f_{10\times i}\}$, that is, the *i*th feature subset contains the top 10 × *i* features in *F*. Then, for each of these feature subsets, the selected classifier was trained and evaluated on the samples that were represented by features in this set using 10-fold cross-validation (Kohavi, 1995; Chen et al., 2018b; Chen et al., 2018d; Guo et al., 2018; Pan et al., 2018; Wang et al., 2018; Zhao et al., 2018; Zhao et al., 2019). According to the results of these feature subsets, a feature number interval [min, max], on which the classifier provided satisfied the prediction performance, can be obtained. The size of the optimal feature set was in this interval with a high probability. In the second stage, based on the above feature number interval [min, max], another series of feature subsets was produced, denoted as $F_{\min}^{2},F_{\min+1}^{2},\ldots ,F_{\max}^{2}$, in which the latter subset contains one more feature than the former one. Similarly, the classifier was trained and evaluated on these subsets, like the first stage. We can obtain a feature subset with the best performance. For convenience, features in this set were still called optimal features, whereas the corresponding classifier was termed as the optimal classifier.

### SVM

As mentioned in the section *Incremental Feature Selection*, the IFS method required a supervised classifier. Here, we selected the classic classifier, SVM (Cortes and Vapnik, 1995). The SVM is a popular supervised learning method that distinguishes samples based on a set of features, and it is widely used to deal with many biological problems (Pan and Shen, 2009; Chen et al., 2017b; Cui and Chen, 2019). The basic principle is to infer a hyperplane with maximum margin between two classes of samples. In reality, most of the data are non-linear in low-dimensional space. In this case, all samples are mapped to a high-dimensional space using kernel function, such as Gaussian kernel. In this space, a linear function can be found to perfectly separate samples of two classes. The original SVM is mainly developed for binary classification. For multi-class classification, the “One Versus the Rest” strategy is adopted. In detail, it constructs *m* binary SVM classifiers for *m* classes, where each classifier is trained to separate samples in one class from the rest using the samples of that class as positive samples and other samples as negative ones. For an unseen sample, *m* probability scores can be yielded by *m* SVM classifiers, and the label with the highest probability score is assigned to the unseen sample.

#### Performance Measurement

For a classification problem with multiple classes, the basic measurement is the individual accuracy for each class, which is defined as

(3) $$ACC_{i}=\frac{M_{i}}{N_{i}}$$

where *ACC* *_i_* represents the individual accuracy of the *i*th class, *M* *_i_* represents the number of correctly predicted samples in the *i*th class, and *N* *_i_* represents the total number of samples in the *i*th class. Furthermore, the overall accuracy can completely evaluate the prediction performance, which is formulated by

(4) $$ACC=\frac{\sum_{i=1}^{8}M_{i}}{\sum_{i=1}^{8}N_{i}}$$

Although the overall accuracy can completely evaluate the prediction quality, it is not a fair measurement when the class sizes are of great difference. According to **Table 1**, the biggest class (“Large intestine”) is about three times as many as the smallest class (“Skin”). In this case, the overall accuracy was not a good choice to assess the prediction quality. Thus, we further employed Matthew’s correlation coefficient (MCC) in multi-class (Gorodkin, 2004). It is a generalization version of MCC proposed by Matthew (Matthews, 1975; Chen et al., 2017a; Zhao et al., 2018; Zhao et al., 2019). It is known that the classic MCC is a balanced measurement even if the class sizes vary greatly. The MCC in multi-class keeps such merit. Suppose we have *n* samples (*i* = 1, 2,…, *n*) and *C* classes (*j* = 1, 2,…, *C*). Let *X* = (*x* *_ij_*)*_n_*_×_ *_C_* be the predicted classes of samples and x*_ij_* ∈{0,1} be a binary value. *x* *_ij_* is equal to 1 if the sample *i* is predicted to belong to class *j*; otherwise, the value *x* *_ij_* is 0. The matrix *Y* = (*y* *_ij_*)*_n_*_×_ *_C_* is defined as the true classes of samples, where the binary variable *y* *_ij_* = 1 means that the sample *i* belongs to class *j*; otherwise, it is set to 0.

According to matrices *X* and *Y*, the MCC can be defined as follows:

(5) $$\begin{array}{l} MCC=\\ \frac{\mathrm{cov}(X,Y)}{\sqrt{\mathrm{cov}(X,X)\mathrm{cov}(Y,Y)}}=\frac{\sum_{i=1}^{n}\sum_{j=1}^{C}\left(x_{ij}-{\bar{x}}_{j})(y_{ij}-{\bar{y}}_{j}\right)}{\sqrt{\sum_{i=1}^{n}\sum_{j=1}^{C}{\left(x_{ij}-{\bar{x}}_{j}\right)}^{2}\sum_{i=1}^{n}\sum_{j=1}^{C}{\left(y_{ij}-{\bar{y}}_{j}\right)}^{2}}}, \end{array}$$

where ${\bar{x}}_{j}$ and ${\bar{y}}_{j}$ are the mean values of members in the *j*-th column of *X* and *j*-th column of *Y*, respectively.

## Results

**Figure 1:**
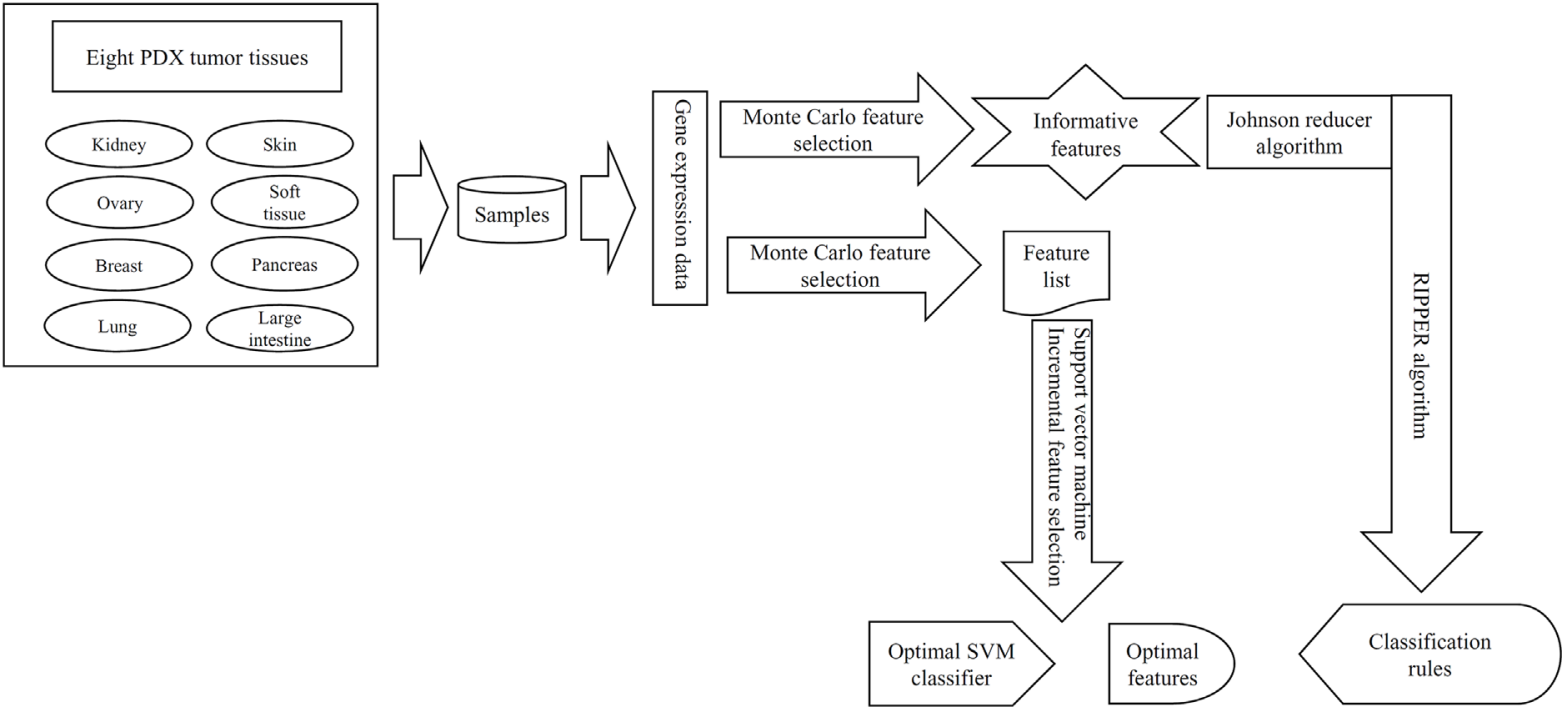
The entire procedures to investigate the gene expression data of samples in eight PDX tumor tissues. These data were first analyzed by the Monte Carlo feature selection method, producing a feature list and informative features. The feature list was used in the incremental feature selection method to extract optimal features for support vector machine (SVM) and construct the optimal SVM classifier. For informative features, the Johnson reducer and Repeated Incremental Pruning to Produce Error Reduction (RIPPER) algorithms were applied on them to generate classification rules.

In this study, a computational investigation on the gene expression data of samples in eight PDX tumor tissues was performed. The entire procedure is illustrated in **Figure 1**.

### Results of MCFS Method

To evaluate the investigated features mentioned in the section *Dataset* on discriminating samples from different tissues, the MCFS method was used to analyze and rank them in descending order according to their RI values. The obtained feature list is provided in **Supplementary Table 1**.

**Figure 2:**
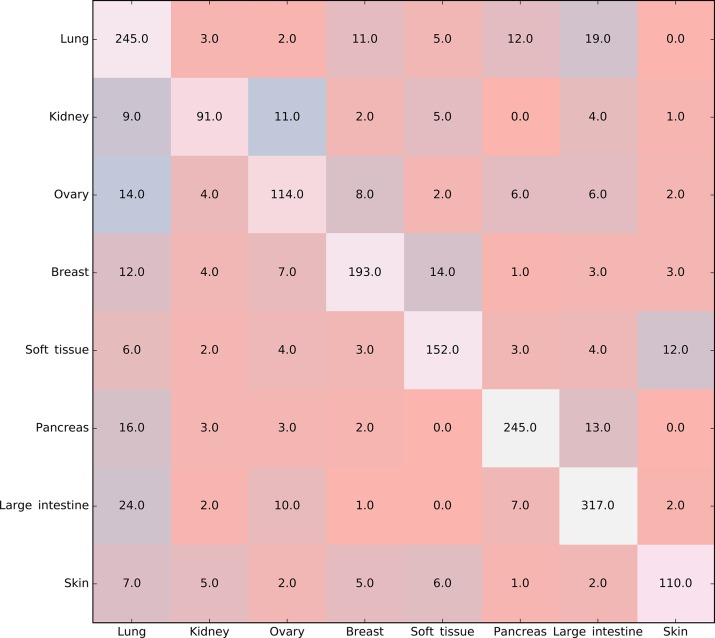
Confusion map for classifying samples into eight tissues *via* the classification rules yielded by Johnson reducer and Repeated Incremental Pruning to Produce Error Reduction (RIPPER) algorithms, evaluated by 10-fold cross-validation thrice.

**Figure 3:**
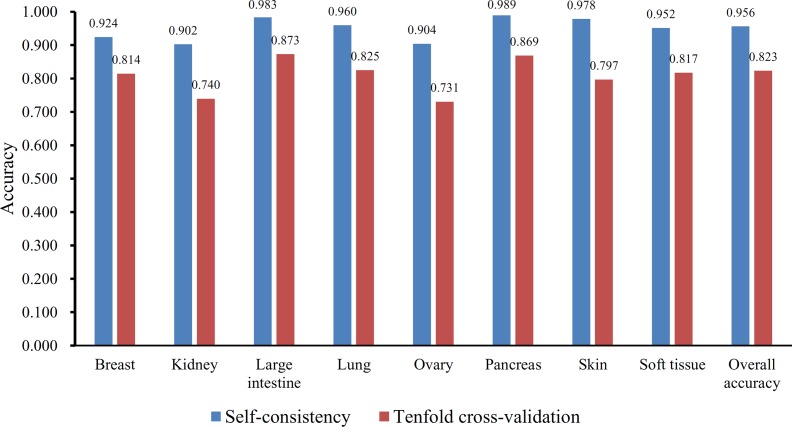
The individual and overall accuracies of the classification rules yielded by Johnson reducer and Repeated Incremental Pruning to Produce Error Reduction (RIPPER) algorithms, evaluated by self-consistency and 10-fold cross-validation.

Furthermore, the MCFS method produced 530 informative features by determining the threshold of RI value as 0.0155. Based on these features, the Johnson reducer and RIPPER algorithms can generate some classification rules. To evaluate the performance of the rules yielded by these two algorithms, 10-fold cross-validation was performed thrice. The confusion map for such test to classify samples into eight tissues is shown in **Figure 2**. The MCC was 0.794. The individual accuracies for eight tissues and overall accuracy are shown in **Figure 3**. It can be seen that the performance of the rules yielded by Johnson reducer and RIPPER algorithms was acceptable. Thus, we further used Johnson reducer and RIPPER algorithms to generate 16 classification rules with 530 informative features based on all samples, which are listed in **Table 2**. The performance of these rules was evaluated by self-consistency; i.e., these rules were applied to samples to make classification. We obtained the MCC of 0.949. The individual and overall accuracies are illustrated in **Figure 3**. It can be observed that the predicted results yielded by self-consistency were much better than those of 10-fold cross-validation. It is reasonable because in self-consistency, samples were classified by the rules generated by themselves.

**Table 2:** Sixteen produced classification rules for distinguishing samples from different tissues.

Rules	Criteria	Tissues
Rule-1	ANGPTL4 ≥ 6.409 BHMT2 ≥ 4.826	Kidney
Rule-2	UPK1A ≥ 6.474	Kidney
Rule-3	PAX3 ≥ 3.401 MIA ≥ 3.562	Skin
Rule-4	BHMT2 ≥ 5.125 ANXA10 ≥ 3.820	Skin
Rule-5	PAX8 ≥ 3.217 ADAM10 ≥ 5.994	Ovary
Rule-6	TRADD ≤ 3.210 ASRGL1 ≥ 6.703	Ovary
Rule-7	CPVL ≥ 7.240 CDX1 ≤ 2.111	Ovary
Rule-8	F11R ≤ 4.935 VSNL1 ≤ 4.528	Soft tissue
Rule-9	HSD17B11≤5.122 ITGA2 ≤ 6.021	Breast
Rule-10	VIM ≥ 8.697 ABHD17C ≥ 3.622	Breast
Rule-11	ADAM28 ≥ 3.637 BTBD6 ≤ 7.581	Pancreas
Rule-12	CXCL5 ≥3.927 PCDH1 ≥ 4.141	Pancreas
Rule-13	LOC102724689 ≥ 7.396	Pancreas
Rule-14	MSN ≥ 5.037 PDGFC ≥ 1.903 BCL2L15 ≤ 5.317	Lung
Rule-15	TP73-AS1 ≥ 3.462 ADAM10 ≥ 6.134	Lung
Rule-16	Other conditions	Large intestine

### Results of the IFS Method

**Figure 4:**
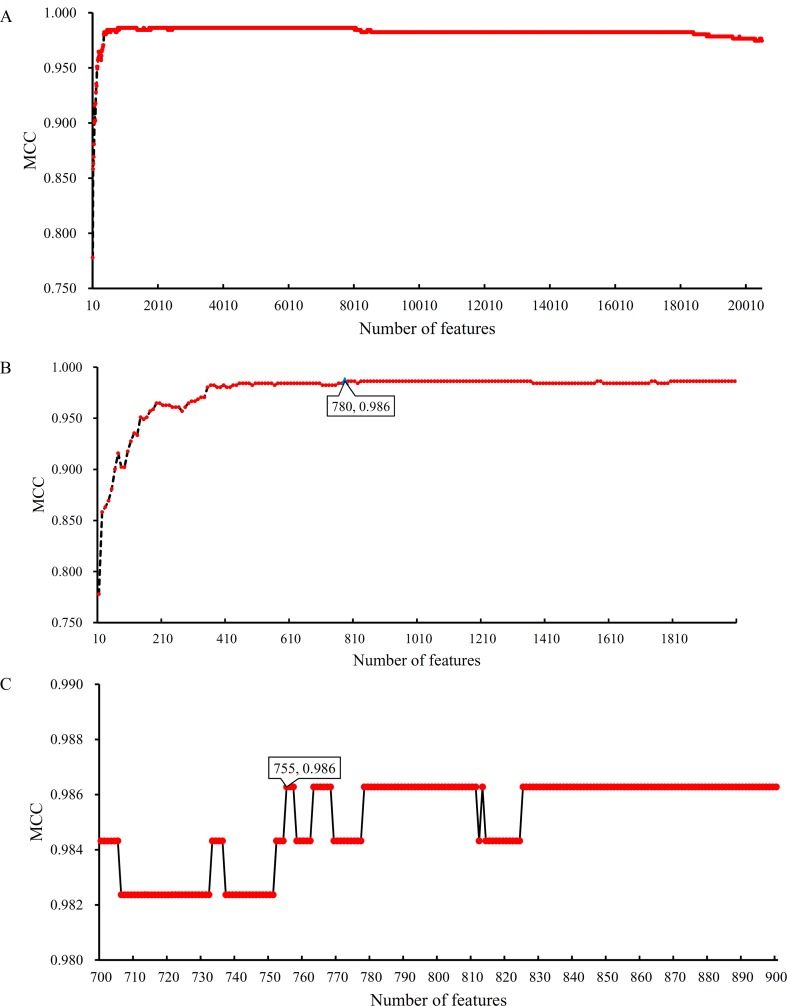
Curves illustrating the performance of SVM on different feature sets. The *X*-axis represents the number of features participating in the classification; the *Y*-axis represents the MCC. **(A)** The whole curve illustrating the performance of SVM on feature sets containing multiples of 10 top features. **(B)** Part of the curve between *X*-axis 10 and 2000. When the top 780 features are used, the MCC reaches the highest (0.986). **(C)** The curve illustrating the performance of SVM on feature sets containing 700–900 top features. When the top 755 features are used, the MCC reaches the highest (0.986).

**Figure 5:**
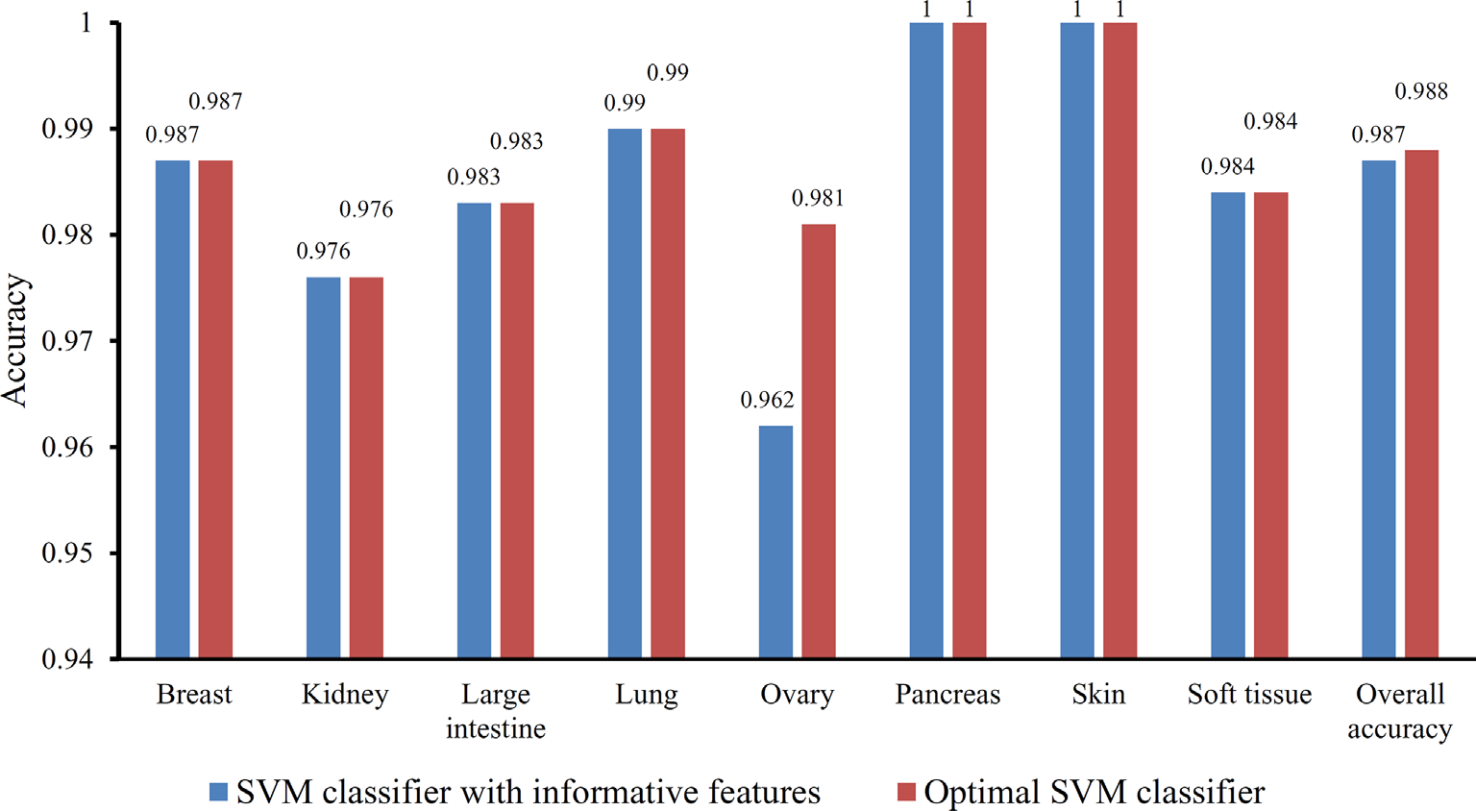
Bar chart illustrating the individual accuracy on each tissue and overall accuracy yielded by the optimal SVM classifier and the classifier with informative features.

Based on the Johnson reducer and RIPPER algorithms, classification rules were generated. However, their performance was not very high. Thus, we further applied SVMs to classify samples from different tissues by integrating the selected features from two-stage IFS method. In the first stage, the feature sets containing multiples of 10 features were constructed, and the SVM was trained on the dataset, in which samples were represented by features in these sets. The 10-fold cross-validation was adopted to evaluate the performance of SVM. The predicted results were counted as individual accuracy for each tissue, overall accuracy, and MCC described in the section *Performance Measurement*, which are provided in **Supplementary Table 2**. For easy observation of the performance of SVM under different feature sets, a curve was plotted in **Figure 4** **A**, in which the number of used features was termed as *X*-axis and MCC as the *Y*-axis. The curve first follows a sharp increasing trend and eventually becomes stable. To clearly illustrate the increasing trend at the beginning of this curve, we plotted the part of the curve between *X*-axis 10 and 2000 in **Figure 4** **B**. The highest MCC is 0.986 when the top 780 features were used. Around 780, the MCCs were also very high. Thus, we determined the feature number interval as [700, 900]. The second stage of the IFS method constructed a second set of feature subsets with a step 1 within feature number interval [700, 900]; that is, all feature sets containing 700–900 features were constructed. SVM and 10-fold cross-validation were adopted to test the discriminating ability of each feature set. The obtained measurements, including individual accuracy for each tissue, overall accuracy, and MCC, are listed in **Supplementary Table 3**. Similarly, we also plotted a curve, as shown in **Figure 4** **C**. The highest MCC is still 0.986; however, it can be achieved only by using the top 755 features. Therefore, these 755 features were termed as optimal features, and the SVM classifier based on these features was the optimal SVM classifier. The detailed performance of such optimal classifier is illustrated in **Figure 5**, from which we can see that all samples in pancreas and skin were correctly classified, and most samples in other tissues were also predicted correctly, indicating the effectiveness of this classifier.

### Superiority of the Optimal Features

**Figure 6:**
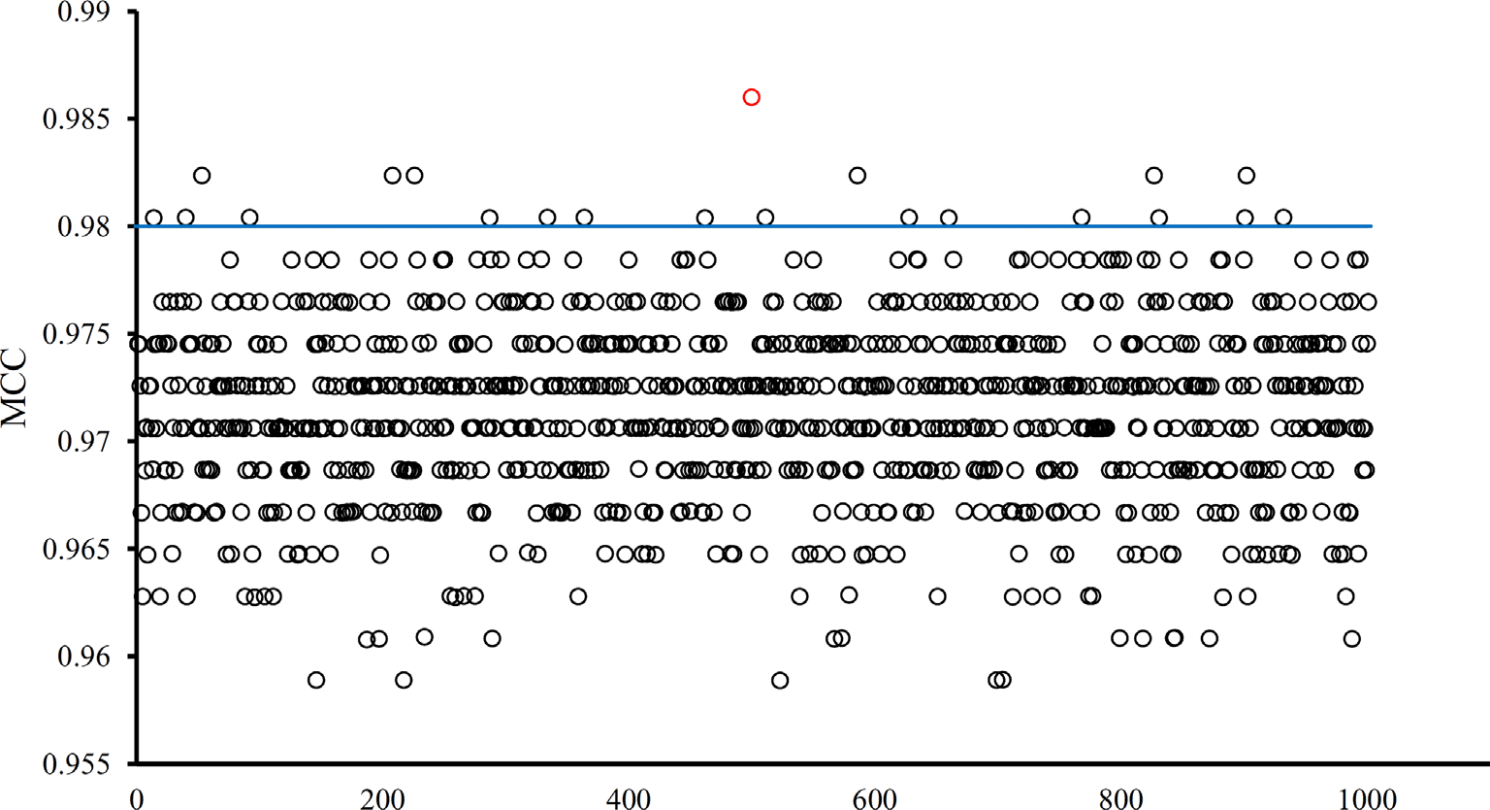
MCCs obtained by the optimal SVM classifier and 1000 SVM classifiers on 1000 randomly generated feature subsets. The red circle represents the MCC yielded by the optimal SVM classifier and black circles represent MCCs produced by SVM classifiers on randomly generated feature subsets. The blue line represents the threshold of high significance level (*p* value < 0.05).

The optimal SVM classifier adopted 755 features to represent samples. To further indicate the importance of these features, we randomly produced 1000 feature subsets, each of which contained 755 features. For each subset, an SVM classifier was constructed, and we evaluated its performance *via* 10-fold cross-validation. The obtained 1000 MCCs are illustrated in **Figure 6** (black circles), in which the MCC yielded by the optimal SVM classifier is also listed (red circle). It can be observed that the MCC yielded by the optimal SVM classifier was higher than all other MCCs. In addition, it was also higher than the threshold of high significance level (*p* value < 0.05), indicating that these 755 features were significant.

Besides, the MCFS method can produce informative features for each given dataset. For our dataset, 530 informative features were obtained. An SVM classifier can be constructed on these features. Such classifier was also evaluated by 10-fold cross-validation. The MCC was 0.984, which was lower than that of the optimal SVM classifier (0.986). The individual accuracies for eight tissues and overall accuracy are illustrated in **Figure 5**, from which we can see that each measurement was no higher than that of the optimal SVM classifier. It is implied that the optimal SVM classifier was superior to the classifier with informative features. The IFS method is useful to extract optimal features for a given classifier.

## Discussion

Based on a new study (Gao et al., 2015) on the expression profile of various tumor subtypes in PDX models, we deeply analyzed this profile for the accurate identification of eight different candidate tumor subtypes using several advanced computational methods in the present study. On the one hand, a list of effective genes that may directly contribute to the qualitative distinction of different tumor subtypes was screened out. On the other hand, we also identified a group of quantitative rules for the accurate identification of each tumor subtypes. This section provides an extensive analysis on the extracted genes and quantitative rules *via* literature reviewing.

### Analysis of Optimal Features (Genes)

For constructing an optimal SVM classifier, the top 755 features (genes) were used to represent samples. However, analyzing them individually is challenging. By carefully checking the performance of SVM classifiers in the first stage of the IFS method, we found that the MCC achieved 0.980 when the top 350 features were used. Thus, we believed that these 350 features were more important than the other 405 features. However, it is still impossible to analyze these 350 features one by one. Here, we selected the most important genes, that is, the top 10 genes, listed in **Table 3**, to provide an extensive analysis.

**Table 3:** Top 10 features (genes) yielded by the MCFS method.

Rank	Gene symbol	Description	RI
1	IFFO1	Intermediate Filament Family Orphan 1	0.4515
2	CDX1	Caudal Type Homeobox 1	0.4263
3	HSD17B11	Hydroxysteroid 17-Beta Dehydrogenase 11	0.4047
4	CHMP4C	Charged Multivesicular Body Protein 4C	0.4042
5	CLIP4	CAP-Gly Domain Containing Linker Protein Family Member 4	0.4025
6	PAX8	Paired Box 8	0.4024
7	GUCY2C	Guanylate Cyclase 2C	0.4023
8	MLANA	Melan-A	0.3857
9	F11R	F11 Receptor	0.3689
10	NR3C1	Nuclear Receptor Subfamily 3 Group C Member 1	0.3646

The top gene is ***IFFO1***, which may have a unique expression pattern in eight tumor tissues. *IFFO1*, encoding a primordial component of the cytoskeleton and nuclear envelope, has been detected with specific methylation patterns and expression profiles in the PDX mouse model of lung cancer (Anglim et al., 2008) and ovarian cancer (Houshdaran et al., 2010), but not in other tumor tissues, indicating that the specific expression pattern of this gene may be a potential biomarker for identifying lung cancer and ovarian cancer.

The gene ***CDX1*** has also been predicted to contribute to distinguishing different PDX tumor tissues at the expression level. With relatively high expression level in small intestine and colon tissues, *CDX1* plays a role in the differentiation of the intestine (Jones et al., 2015). As for its expression in different PDX tumor tissues, this gene has relatively high expression in large intestine-associated tumor tissues of PDX mouse model, confirming the potential distinguishing effect of such gene (Rankin et al., 2004).

***HSD17B11***, encoding short-chain alcohol dehydrogenases, has been widely reported to participate in androgen metabolism during steroidogenesis (Rotinen et al., 2011). As for its contribution on tumorigenesis and specific role during PDX implantation, this gene has only been identified in both primary and implanted tumor tissue of the prostate (Hilborn et al., 2017) and breast tumorigenesis (Rotinen et al., 2011), implying that such gene may distinguish different tumor tissues.

***CHMP4C*** is reported to be involved in multi-vesicular body formation and endosomal cargo sorting (Yu et al., 2009). As for its specific expression pattern in different tumor tissues, this gene has a unique pathological expression profile in multiple tumors of the urine system, implying that CHMP4C may be an effective marker for identifying kidney-associated tumor from other tumor subtypes derived from other tissues (Fujita et al., 2017).

***CLIP4***, encoding one of the components of the cytoplasmic linker protein family, participates in regulating the cellular compartmentalization of the AKT kinase family involved in tumorigenesis (Saber et al., 2016). Such gene has been confirmed to have a unique expression pattern in various tumor PDX mouse models, including clear cell renal cell carcinomas (kidney) (Ahn et al., 2016), lung adenocarcinoma (lung) (Saber et al., 2016), and gastric cancer (stomach) (Chong et al., 2014), implying that this gene may be a biomarker for some tumor subtypes investigated in this study.

***PAX8***, encoding a transcription factor of the paired box (PAX) family, has been predicted to be a potential identification marker for the distinction of different tumor tissues in PDX mouse models (Narumi et al., 2010). Recent studies (Butler et al., 2017) confirmed that the overexpression of such gene may directly induce the initiation and progression of ovarian cancer in PDX mouse models, distinguishing tumorigenesis of such tissue from the other seven tumor tissues.

***GUCY2C***, encoding a membrane-associated guanylate kinase, participates in immune regulation, including T-cell receptor-mediated T-cell activation and proliferation (Snook et al., 2012). As for its tissue-specific distribution in the PDX mouse model, recent studies (Witek et al., 2014) confirmed that in the large intestine (especially colon tissue), the high expression level of such gene in the PDX model indicates that such mouse model was implanted with an invasive large intestine-associated tumor subtype.

The next gene ***MLANA*** encodes a GPR143-associated functional protein contributing to the maintenance of expression, stability, trafficking, and processing of melanocyte protein PMEL (Witek et al., 2014). As for its relationship with different tumor tissues in the PDX mouse model, a recent study (Hollingshead et al., 2014) confirmed that such gene may distinguish melanoma and various skin-derived tumor subtypes in the PDX mouse model from the other seven tumor subtypes.

***F11R***, as a regulator of cell-to-cell adhesion in epithelial cell sheets, has been reported to encode a multi-functional protein that interacts with reovirus (Birse et al., 2017), integrin LFA1 (Gerhardt and Ley, 2015), and platelets (Kedees et al., 2005). As for its distinctive function for different PDX tumor tissues, recent studies (Jansen et al., 2009) confirmed that in the PDX models of glioblastoma (soft-tissue-derived tumorigenesis), F11R has a unique expression pattern compared with other tumor tissues.

***NR3C1***, encoding a tissue-specific transcriptional activator, has been widely reported to be involved in chromatin remodeling (Geng et al., 2016) and cell proliferation in tissues *in situ* (Souza et al., 2014). As for its distinctive expression pattern in different tumor tissues, such gene has a relatively high expression pattern in various tumor subtypes, including lung cancer (Lajoie et al., 2014) and kidney cancer (Zaravinos et al., 2014), compared with other tumor subtypes.

Overall, based on advanced computational methods, we screened out a group of effective tumor-associated genes that may distinguish different tumor subtypes from PDX mouse models. From the discussions on the top 10 genes, we confirmed that other optimal features (genes) may also be important biomarkers for distinguishing different tumor subtypes that need further investigation.

### Analysis of Classification Rules

**Figure 7:**
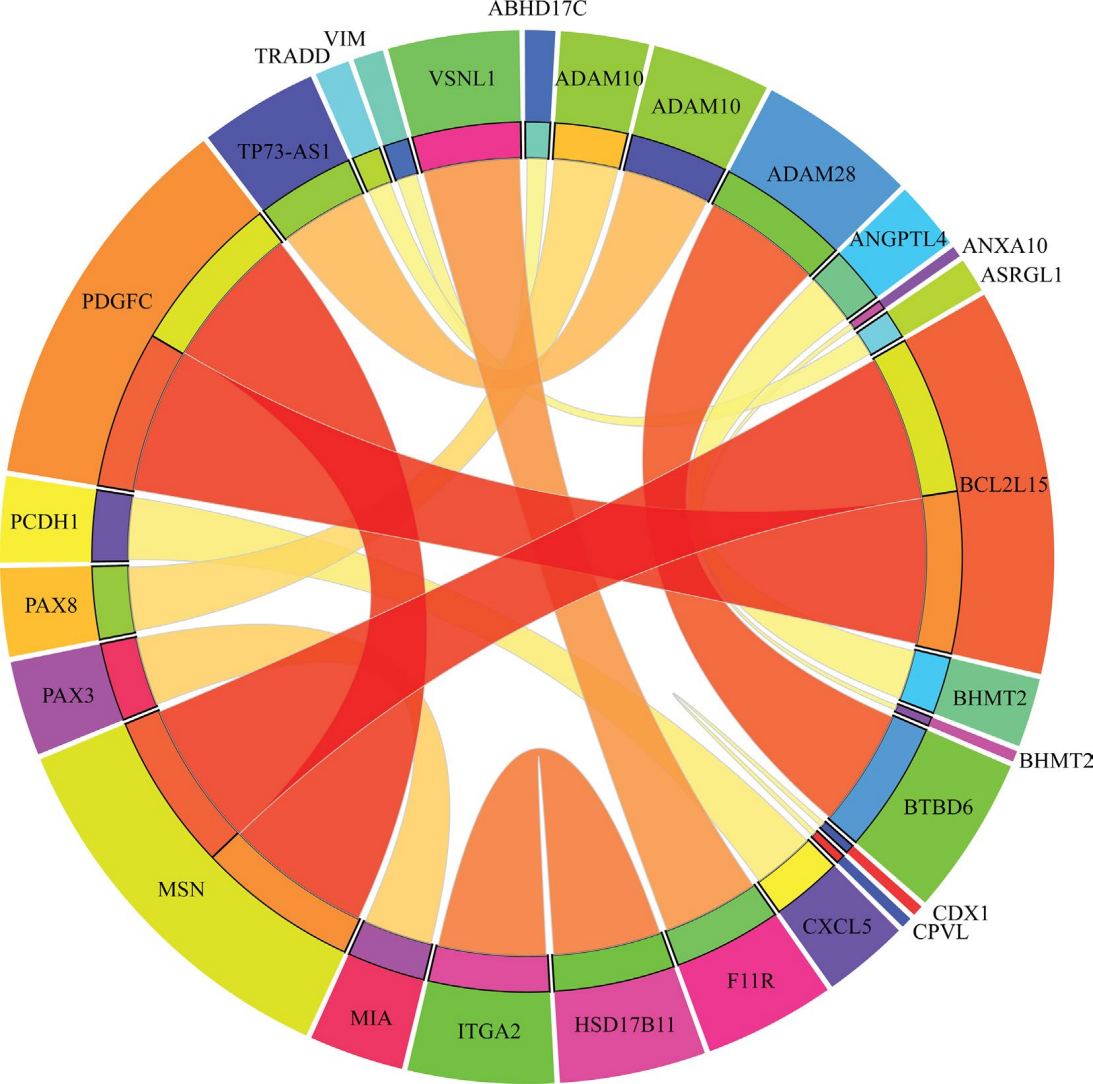
Rule networks for 16 classification rules generated by Ciruvis.

Apart from qualitative biomarkers to distinguish different tumor subtypes in the PDX mouse model, we also summarized 16 classification rules for further quantitative analysis. To show the inner relationship between genes involved in these rules, we draw a rule network *via* Ciruvis (Bornelov et al., 2014), which is illustrated in **Figure 7**. Based on the detailed expression profile data in other similar studies, most of the 16 rules can be confirmed by their rationalities, reflecting the relative expression pattern of such genes involving the rules. The detailed analysis on each rule is shown below.

The first two rules are for the identification of PDX tumor tissues originating from kidney-associated tumor. According to these two quantitative rules, *ANGPTL4* should have higher expression pattern and the expression level of *BHMT2* and *UPK1A* should also be up-regulated. According to recent single-cell RNA sequencing data of the PDX mouse model (Zhu et al., 2017), the expression patterns of the three genes have all been confirmed to have corresponding expression level.

The following two rules are for the identification of skin-derived PDX tumor tissues. Four genes named *PAX3*, *MIA*, *BHMT2*, and *ANXA10* have been screened out as potential parameters for the identification of skin-associated PDX tumors. Based on recent sequencing publications, all four genes have been reported to be upregulated, conforming to these rules (Tso et al., 2014). The combination of such four parameters may improve the efficacy and accuracy for the quantitative identification of skin-derived tumor-implanted PDX mouse model. As for the detailed FPKM value, the dataset provided by similar studies (Wyatt et al., 2014) also corresponds with our rules.

The next three rules describe the expression pattern of ovarian cancer. As we have analyzed above, *PAX8*, encoding a functional transcription factor, has a uniquely high expression pattern in ovarian-cancer-derived PDX tumor tissues, corresponding with Rule-5 (Narumi et al., 2010). As for the other five parameters, a recent study (Dobbin et al., 2014) revealed the specific expression pattern of ovarian cancer after screening the PDX mouse microenvironment. According to recent literature, although the expression profile of *CDX1* (as one of the parameters mentioned above) cannot indicate ovarian cancer alone, the combination of *CDX1* and *CPVL* may be specifically enough to recognize ovarian-tumor-derived PDX mouse tumor tissues (Dobbin et al., 2014). According to the dataset provided by such study, the remaining four parameters (*ADAM10*, *TRADD*, *ASRGL1*, and *CPVL*) have also been validated to basically match our rules.

Only one rule involving two genes may contribute to the identification of soft-tissue-derived PDX tumor tissues. *F11R*, as we have analyzed above, has been confirmed to have a relatively low expression pattern in the PDX tumor tissue derived from soft tissue, which is somewhat different from those derived from other tissues, validating the accuracy and efficacy of this rule (Jansen et al., 2009). A similar expression pattern has also been identified for the remaining soft-tissue-specific expressing gene *VSNL1* (Sarver et al., 2015), corresponding with this rule.

The following two rules contribute to the identification of breast cancer in the PDX mouse model. Four genes, namely, *HSD17B11*, *ITGA2*, *VIM*, and *ABHD17C*, are involved in these rules. The low expression of *HSD17B11* and *ITGA2* and the high expression of *VIM* and *ABHD17C* have all been validated by recent sequencing studies on breast cancer (Rotinen et al., 2011), reflecting the accuracy of these two rules.

The expression levels of five genes (*ADAM28*, *BTBD6*, *CXCL5*, *PCDH1*, and *LOC102724689*) comprise three rules for the identification of pancreatic-tissue-derived PDX tumor tissues. According to another dataset (Martinez-Garcia et al., 2014), the quantitative parameter of such five genes have been basically validated. Among such five genes, *PCDH1* is the most effective tumor-associated gene, contributing to pancreatic cancer with abnormal promoter methylation status and participating in FGFR-associated signaling pathways (Zhang et al., 2014).

The two remaining rules contribute to the identification of lung-tissue-derived PDX tumor tissues. Five genes, namely, *MSN*, *PDGFC*, *BCL2L15*, *TP73-AS1*, and *ADAM10*, were screened out as candidate parameters. Various studies have revealed the expression pattern of lung cancer in PDX mouse model at either the single cell or bullet level (Bradford et al., 2016). By comprehensively analyzing such expression profiles of the five candidate genes, the expression levels of such five genes in lung-cancer-derived PDX tumor tissues correspond to the quantitative rules. Furthermore, if the expression profile of a certain PDX tumor tissue does not satisfy any of the conditions we mentioned above, such PDX tumor tissue may be derived from the large intestine.

Overall, we quantitatively analyzed the 16 rules reported in this study. Several rules can be supported or validated by recent RNA sequencing datasets on PDX tumor tissues, validating the efficacy and accuracy of these rules. Combining the qualitative analysis presented in the section *Analysis of Optimal Features (Genes)*, we not only identified a group of highly related PDX tumor-specific biomarkers at the expression spectrum level but also for the first time attempted to build a systematic distinctive standard for the quantitative identification of PDX tumor originating from different tissue subtypes. The genes and rules that we screened out not only can provide a new tool for the identification of PDX-derived tumors originating from different primary tissues but also reveal the distinctive expression characteristics and expression profile stability of PDX-derived tumor tissues compared with the primary ones, validating the efficacy and practicability of the PDX mouse model in tumor studies.

## Data Availability

Publicly available datasets were analyzed in this study. This data can be found here: https://www.ncbi.nlm.nih.gov/geo/query/acc.cgi?acc=GSE78806

## Author Contributions

All authors contributed to the research and reviewed the manuscript. TH and YDC designed the study. LC, XP, and KYF performed the experiments. YHZ and XH analyzed the results. LC and XP wrote the manuscript.

## Funding

This study was funded by the National Natural Science Foundation of China (31701151), the Natural Science Foundation of Shanghai (17ZR1412500), the National Key R&D Program of China (2018YFC0910403), the Shanghai Sailing Program (16YF1413800), the Youth Innovation Promotion Association of Chinese Academy of Sciences (CAS) (2016245), the fund of the key Laboratory of Stem Cell Biology of Chinese Academy of Sciences (201703), and the Science and Technology Commission of Shanghai Municipality (STCSM) (18dz2271000).

## Conflict of Interest Statement

The authors declare that the research was conducted in the absence of any commercial or financial relationships that could be construed as a potential conflict of interest.

The reviewer QZ declared a past co-authorship with one of the authors LC to the handling editor.
