# Supplementary material for: Primary Tumor Site Specificity is Preserved in Patient-Derived Tumor Xenograft Models
Source: Front Genet. 2019 Aug 13;10:738. doi: 10.3389/fgene.2019.00738 (PMC6701289; doi:10.3389/fgene.2019.00738)
Supplement: Supplementary file 3 [file Table_3.docx]

**Supplementary Table 3.** The performance of SVM on different feature sets that contain 700-900 features.

| **Number of features** | **Accuracy for breast** | **Accuracy for kidney** | **Accuracy for large intestine** | **Accuracy for lung** | **Accuracy for ovary** | **Accuracy for pancreas** | **Accuracy for skin** | **Accuracy for soft tissue** | **Overall accuracy** | **MCC** |
| --- | --- | --- | --- | --- | --- | --- | --- | --- | --- | --- |
| 700 | 0.987 | 0.976 | 0.983 | 0.990 | 0.962 | 1.000 | 1.000 | 0.984 | 0.987 | 0.984 |
| 701 | 0.987 | 0.976 | 0.983 | 0.990 | 0.962 | 1.000 | 1.000 | 0.984 | 0.987 | 0.984 |
| 702 | 0.987 | 0.976 | 0.983 | 0.990 | 0.962 | 1.000 | 1.000 | 0.984 | 0.987 | 0.984 |
| 703 | 0.987 | 0.976 | 0.983 | 0.990 | 0.962 | 1.000 | 1.000 | 0.984 | 0.987 | 0.984 |
| 704 | 0.987 | 0.976 | 0.983 | 0.990 | 0.962 | 1.000 | 1.000 | 0.984 | 0.987 | 0.984 |
| 705 | 0.987 | 0.976 | 0.983 | 0.990 | 0.962 | 1.000 | 1.000 | 0.984 | 0.987 | 0.984 |
| 706 | 0.987 | 0.976 | 0.983 | 0.990 | 0.942 | 1.000 | 1.000 | 0.984 | 0.985 | 0.982 |
| 707 | 0.987 | 0.976 | 0.983 | 0.990 | 0.942 | 1.000 | 1.000 | 0.984 | 0.985 | 0.982 |
| 708 | 0.987 | 0.976 | 0.983 | 0.990 | 0.942 | 1.000 | 1.000 | 0.984 | 0.985 | 0.982 |
| 709 | 0.987 | 0.976 | 0.983 | 0.990 | 0.942 | 1.000 | 1.000 | 0.984 | 0.985 | 0.982 |
| 710 | 0.987 | 0.976 | 0.983 | 0.990 | 0.942 | 1.000 | 1.000 | 0.984 | 0.985 | 0.982 |
| 711 | 0.987 | 0.976 | 0.983 | 0.990 | 0.942 | 1.000 | 1.000 | 0.984 | 0.985 | 0.982 |
| 712 | 0.987 | 0.976 | 0.983 | 0.990 | 0.942 | 1.000 | 1.000 | 0.984 | 0.985 | 0.982 |
| 713 | 0.987 | 0.976 | 0.983 | 0.990 | 0.942 | 1.000 | 1.000 | 0.984 | 0.985 | 0.982 |
| 714 | 0.987 | 0.976 | 0.983 | 0.990 | 0.942 | 1.000 | 1.000 | 0.984 | 0.985 | 0.982 |
| 715 | 0.987 | 0.976 | 0.983 | 0.990 | 0.942 | 1.000 | 1.000 | 0.984 | 0.985 | 0.982 |
| 716 | 0.987 | 0.976 | 0.983 | 0.990 | 0.942 | 1.000 | 1.000 | 0.984 | 0.985 | 0.982 |
| 717 | 0.987 | 0.976 | 0.983 | 0.990 | 0.942 | 1.000 | 1.000 | 0.984 | 0.985 | 0.982 |
| 718 | 0.987 | 0.976 | 0.983 | 0.990 | 0.942 | 1.000 | 1.000 | 0.984 | 0.985 | 0.982 |
| 719 | 0.987 | 0.976 | 0.983 | 0.990 | 0.942 | 1.000 | 1.000 | 0.984 | 0.985 | 0.982 |
| 720 | 0.987 | 0.976 | 0.983 | 0.990 | 0.942 | 1.000 | 1.000 | 0.984 | 0.985 | 0.982 |
| 721 | 0.987 | 0.976 | 0.983 | 0.990 | 0.942 | 1.000 | 1.000 | 0.984 | 0.985 | 0.982 |
| 722 | 0.987 | 0.976 | 0.983 | 0.990 | 0.942 | 1.000 | 1.000 | 0.984 | 0.985 | 0.982 |
| 723 | 0.987 | 0.976 | 0.983 | 0.990 | 0.942 | 1.000 | 1.000 | 0.984 | 0.985 | 0.982 |
| 724 | 0.987 | 0.976 | 0.983 | 0.990 | 0.942 | 1.000 | 1.000 | 0.984 | 0.985 | 0.982 |
| 725 | 0.987 | 0.976 | 0.983 | 0.990 | 0.942 | 1.000 | 1.000 | 0.984 | 0.985 | 0.982 |
| 726 | 0.987 | 0.976 | 0.983 | 0.990 | 0.942 | 1.000 | 1.000 | 0.984 | 0.985 | 0.982 |
| 727 | 0.987 | 0.976 | 0.983 | 0.990 | 0.942 | 1.000 | 1.000 | 0.984 | 0.985 | 0.982 |
| 728 | 0.987 | 0.976 | 0.983 | 0.990 | 0.942 | 1.000 | 1.000 | 0.984 | 0.985 | 0.982 |
| 729 | 0.987 | 0.976 | 0.983 | 0.990 | 0.942 | 1.000 | 1.000 | 0.984 | 0.985 | 0.982 |
| 730 | 0.987 | 0.976 | 0.983 | 0.990 | 0.942 | 1.000 | 1.000 | 0.984 | 0.985 | 0.982 |
| 731 | 0.987 | 0.976 | 0.983 | 0.990 | 0.942 | 1.000 | 1.000 | 0.984 | 0.985 | 0.982 |
| 732 | 0.987 | 0.976 | 0.983 | 0.990 | 0.942 | 1.000 | 1.000 | 0.984 | 0.985 | 0.982 |
| 733 | 0.987 | 0.976 | 0.983 | 0.990 | 0.962 | 1.000 | 1.000 | 0.984 | 0.987 | 0.984 |
| 734 | 0.987 | 0.976 | 0.983 | 0.990 | 0.962 | 1.000 | 1.000 | 0.984 | 0.987 | 0.984 |
| 735 | 0.987 | 0.976 | 0.983 | 0.990 | 0.962 | 1.000 | 1.000 | 0.984 | 0.987 | 0.984 |
| 736 | 0.987 | 0.976 | 0.983 | 0.990 | 0.962 | 1.000 | 1.000 | 0.984 | 0.987 | 0.984 |
| 737 | 0.987 | 0.976 | 0.983 | 0.990 | 0.942 | 1.000 | 1.000 | 0.984 | 0.985 | 0.982 |
| 738 | 0.987 | 0.976 | 0.983 | 0.990 | 0.942 | 1.000 | 1.000 | 0.984 | 0.985 | 0.982 |
| 739 | 0.987 | 0.976 | 0.983 | 0.990 | 0.942 | 1.000 | 1.000 | 0.984 | 0.985 | 0.982 |
| 740 | 0.987 | 0.976 | 0.983 | 0.990 | 0.942 | 1.000 | 1.000 | 0.984 | 0.985 | 0.982 |
| 741 | 0.987 | 0.976 | 0.983 | 0.990 | 0.942 | 1.000 | 1.000 | 0.984 | 0.985 | 0.982 |
| 742 | 0.987 | 0.976 | 0.983 | 0.990 | 0.942 | 1.000 | 1.000 | 0.984 | 0.985 | 0.982 |
| 743 | 0.987 | 0.976 | 0.983 | 0.990 | 0.942 | 1.000 | 1.000 | 0.984 | 0.985 | 0.982 |
| 744 | 0.987 | 0.976 | 0.983 | 0.990 | 0.942 | 1.000 | 1.000 | 0.984 | 0.985 | 0.982 |
| 745 | 0.987 | 0.976 | 0.983 | 0.990 | 0.942 | 1.000 | 1.000 | 0.984 | 0.985 | 0.982 |
| 746 | 0.987 | 0.976 | 0.983 | 0.990 | 0.942 | 1.000 | 1.000 | 0.984 | 0.985 | 0.982 |
| 747 | 0.987 | 0.976 | 0.983 | 0.990 | 0.942 | 1.000 | 1.000 | 0.984 | 0.985 | 0.982 |
| 748 | 0.987 | 0.976 | 0.983 | 0.990 | 0.942 | 1.000 | 1.000 | 0.984 | 0.985 | 0.982 |
| 749 | 0.987 | 0.976 | 0.983 | 0.990 | 0.942 | 1.000 | 1.000 | 0.984 | 0.985 | 0.982 |
| 750 | 0.987 | 0.976 | 0.983 | 0.990 | 0.942 | 1.000 | 1.000 | 0.984 | 0.985 | 0.982 |
| 751 | 0.987 | 0.976 | 0.983 | 0.990 | 0.942 | 1.000 | 1.000 | 0.984 | 0.985 | 0.982 |
| 752 | 0.987 | 0.976 | 0.983 | 0.990 | 0.962 | 1.000 | 1.000 | 0.984 | 0.987 | 0.984 |
| 753 | 0.987 | 0.976 | 0.983 | 0.990 | 0.962 | 1.000 | 1.000 | 0.984 | 0.987 | 0.984 |
| 754 | 0.987 | 0.976 | 0.983 | 0.990 | 0.962 | 1.000 | 1.000 | 0.984 | 0.987 | 0.984 |
| 755 | 0.987 | 0.976 | 0.983 | 0.990 | 0.981 | 1.000 | 1.000 | 0.984 | 0.988 | 0.986 |
| 756 | 0.987 | 0.976 | 0.983 | 0.990 | 0.981 | 1.000 | 1.000 | 0.984 | 0.988 | 0.986 |
| 757 | 0.987 | 0.976 | 0.983 | 0.990 | 0.981 | 1.000 | 1.000 | 0.984 | 0.988 | 0.986 |
| 758 | 0.987 | 0.976 | 0.983 | 0.990 | 0.962 | 1.000 | 1.000 | 0.984 | 0.987 | 0.984 |
| 759 | 0.987 | 0.976 | 0.983 | 0.990 | 0.962 | 1.000 | 1.000 | 0.984 | 0.987 | 0.984 |
| 760 | 0.987 | 0.976 | 0.983 | 0.990 | 0.962 | 1.000 | 1.000 | 0.984 | 0.987 | 0.984 |
| 761 | 0.987 | 0.976 | 0.983 | 0.990 | 0.962 | 1.000 | 1.000 | 0.984 | 0.987 | 0.984 |
| 762 | 0.987 | 0.976 | 0.983 | 0.990 | 0.962 | 1.000 | 1.000 | 0.984 | 0.987 | 0.984 |
| 763 | 0.987 | 0.976 | 0.983 | 0.990 | 0.981 | 1.000 | 1.000 | 0.984 | 0.988 | 0.986 |
| 764 | 0.987 | 0.976 | 0.983 | 0.990 | 0.981 | 1.000 | 1.000 | 0.984 | 0.988 | 0.986 |
| 765 | 0.987 | 0.976 | 0.983 | 0.990 | 0.981 | 1.000 | 1.000 | 0.984 | 0.988 | 0.986 |
| 766 | 0.987 | 0.976 | 0.983 | 0.990 | 0.981 | 1.000 | 1.000 | 0.984 | 0.988 | 0.986 |
| 767 | 0.987 | 0.976 | 0.983 | 0.990 | 0.981 | 1.000 | 1.000 | 0.984 | 0.988 | 0.986 |
| 768 | 0.987 | 0.976 | 0.983 | 0.990 | 0.981 | 1.000 | 1.000 | 0.984 | 0.988 | 0.986 |
| 769 | 0.987 | 0.976 | 0.983 | 0.990 | 0.962 | 1.000 | 1.000 | 0.984 | 0.987 | 0.984 |
| 770 | 0.987 | 0.976 | 0.983 | 0.990 | 0.962 | 1.000 | 1.000 | 0.984 | 0.987 | 0.984 |
| 771 | 0.987 | 0.976 | 0.983 | 0.990 | 0.962 | 1.000 | 1.000 | 0.984 | 0.987 | 0.984 |
| 772 | 0.987 | 0.976 | 0.983 | 0.990 | 0.962 | 1.000 | 1.000 | 0.984 | 0.987 | 0.984 |
| 773 | 0.987 | 0.976 | 0.983 | 0.990 | 0.962 | 1.000 | 1.000 | 0.984 | 0.987 | 0.984 |
| 774 | 0.987 | 0.976 | 0.983 | 0.990 | 0.962 | 1.000 | 1.000 | 0.984 | 0.987 | 0.984 |
| 775 | 0.987 | 0.976 | 0.983 | 0.990 | 0.962 | 1.000 | 1.000 | 0.984 | 0.987 | 0.984 |
| 776 | 0.987 | 0.976 | 0.983 | 0.990 | 0.962 | 1.000 | 1.000 | 0.984 | 0.987 | 0.984 |
| 777 | 0.987 | 0.976 | 0.983 | 0.990 | 0.962 | 1.000 | 1.000 | 0.984 | 0.987 | 0.984 |
| 778 | 0.987 | 0.976 | 0.983 | 0.990 | 0.981 | 1.000 | 1.000 | 0.984 | 0.988 | 0.986 |
| 779 | 0.987 | 0.976 | 0.983 | 0.990 | 0.981 | 1.000 | 1.000 | 0.984 | 0.988 | 0.986 |
| 780 | 0.987 | 0.976 | 0.983 | 0.990 | 0.981 | 1.000 | 1.000 | 0.984 | 0.988 | 0.986 |
| 781 | 0.987 | 0.976 | 0.983 | 0.990 | 0.981 | 1.000 | 1.000 | 0.984 | 0.988 | 0.986 |
| 782 | 0.987 | 0.976 | 0.983 | 0.990 | 0.981 | 1.000 | 1.000 | 0.984 | 0.988 | 0.986 |
| 783 | 0.987 | 0.976 | 0.983 | 0.990 | 0.981 | 1.000 | 1.000 | 0.984 | 0.988 | 0.986 |
| 784 | 0.987 | 0.976 | 0.983 | 0.990 | 0.981 | 1.000 | 1.000 | 0.984 | 0.988 | 0.986 |
| 785 | 0.987 | 0.976 | 0.983 | 0.990 | 0.981 | 1.000 | 1.000 | 0.984 | 0.988 | 0.986 |
| 786 | 0.987 | 0.976 | 0.983 | 0.990 | 0.981 | 1.000 | 1.000 | 0.984 | 0.988 | 0.986 |
| 787 | 0.987 | 0.976 | 0.983 | 0.990 | 0.981 | 1.000 | 1.000 | 0.984 | 0.988 | 0.986 |
| 788 | 0.987 | 0.976 | 0.983 | 0.990 | 0.981 | 1.000 | 1.000 | 0.984 | 0.988 | 0.986 |
| 789 | 0.987 | 0.976 | 0.983 | 0.990 | 0.981 | 1.000 | 1.000 | 0.984 | 0.988 | 0.986 |
| 790 | 0.987 | 0.976 | 0.983 | 0.990 | 0.981 | 1.000 | 1.000 | 0.984 | 0.988 | 0.986 |
| 791 | 0.987 | 0.976 | 0.983 | 0.990 | 0.981 | 1.000 | 1.000 | 0.984 | 0.988 | 0.986 |
| 792 | 0.987 | 0.976 | 0.983 | 0.990 | 0.981 | 1.000 | 1.000 | 0.984 | 0.988 | 0.986 |
| 793 | 0.987 | 0.976 | 0.983 | 0.990 | 0.981 | 1.000 | 1.000 | 0.984 | 0.988 | 0.986 |
| 794 | 0.987 | 0.976 | 0.983 | 0.990 | 0.981 | 1.000 | 1.000 | 0.984 | 0.988 | 0.986 |
| 795 | 0.987 | 0.976 | 0.983 | 0.990 | 0.981 | 1.000 | 1.000 | 0.984 | 0.988 | 0.986 |
| 796 | 0.987 | 0.976 | 0.983 | 0.990 | 0.981 | 1.000 | 1.000 | 0.984 | 0.988 | 0.986 |
| 797 | 0.987 | 0.976 | 0.983 | 0.990 | 0.981 | 1.000 | 1.000 | 0.984 | 0.988 | 0.986 |
| 798 | 0.987 | 0.976 | 0.983 | 0.990 | 0.981 | 1.000 | 1.000 | 0.984 | 0.988 | 0.986 |
| 799 | 0.987 | 0.976 | 0.983 | 0.990 | 0.981 | 1.000 | 1.000 | 0.984 | 0.988 | 0.986 |
| 800 | 0.987 | 0.976 | 0.983 | 0.990 | 0.981 | 1.000 | 1.000 | 0.984 | 0.988 | 0.986 |
| 801 | 0.987 | 0.976 | 0.983 | 0.990 | 0.981 | 1.000 | 1.000 | 0.984 | 0.988 | 0.986 |
| 802 | 0.987 | 0.976 | 0.983 | 0.990 | 0.981 | 1.000 | 1.000 | 0.984 | 0.988 | 0.986 |
| 803 | 0.987 | 0.976 | 0.983 | 0.990 | 0.981 | 1.000 | 1.000 | 0.984 | 0.988 | 0.986 |
| 804 | 0.987 | 0.976 | 0.983 | 0.990 | 0.981 | 1.000 | 1.000 | 0.984 | 0.988 | 0.986 |
| 805 | 0.987 | 0.976 | 0.983 | 0.990 | 0.981 | 1.000 | 1.000 | 0.984 | 0.988 | 0.986 |
| 806 | 0.987 | 0.976 | 0.983 | 0.990 | 0.981 | 1.000 | 1.000 | 0.984 | 0.988 | 0.986 |
| 807 | 0.987 | 0.976 | 0.983 | 0.990 | 0.981 | 1.000 | 1.000 | 0.984 | 0.988 | 0.986 |
| 808 | 0.987 | 0.976 | 0.983 | 0.990 | 0.981 | 1.000 | 1.000 | 0.984 | 0.988 | 0.986 |
| 809 | 0.987 | 0.976 | 0.983 | 0.990 | 0.981 | 1.000 | 1.000 | 0.984 | 0.988 | 0.986 |
| 810 | 0.987 | 0.976 | 0.983 | 0.990 | 0.981 | 1.000 | 1.000 | 0.984 | 0.988 | 0.986 |
| 811 | 0.987 | 0.976 | 0.983 | 0.990 | 0.981 | 1.000 | 1.000 | 0.984 | 0.988 | 0.986 |
| 812 | 0.987 | 0.976 | 0.983 | 0.990 | 0.962 | 1.000 | 1.000 | 0.984 | 0.987 | 0.984 |
| 813 | 0.987 | 0.976 | 0.983 | 0.990 | 0.981 | 1.000 | 1.000 | 0.984 | 0.988 | 0.986 |
| 814 | 0.987 | 0.976 | 0.983 | 0.990 | 0.962 | 1.000 | 1.000 | 0.984 | 0.987 | 0.984 |
| 815 | 0.987 | 0.976 | 0.983 | 0.990 | 0.962 | 1.000 | 1.000 | 0.984 | 0.987 | 0.984 |
| 816 | 0.987 | 0.976 | 0.983 | 0.990 | 0.962 | 1.000 | 1.000 | 0.984 | 0.987 | 0.984 |
| 817 | 0.987 | 0.976 | 0.983 | 0.990 | 0.962 | 1.000 | 1.000 | 0.984 | 0.987 | 0.984 |
| 818 | 0.987 | 0.976 | 0.983 | 0.990 | 0.962 | 1.000 | 1.000 | 0.984 | 0.987 | 0.984 |
| 819 | 0.987 | 0.976 | 0.983 | 0.990 | 0.962 | 1.000 | 1.000 | 0.984 | 0.987 | 0.984 |
| 820 | 0.987 | 0.976 | 0.983 | 0.990 | 0.962 | 1.000 | 1.000 | 0.984 | 0.987 | 0.984 |
| 821 | 0.987 | 0.976 | 0.983 | 0.990 | 0.962 | 1.000 | 1.000 | 0.984 | 0.987 | 0.984 |
| 822 | 0.987 | 0.976 | 0.983 | 0.990 | 0.962 | 1.000 | 1.000 | 0.984 | 0.987 | 0.984 |
| 823 | 0.987 | 0.976 | 0.983 | 0.990 | 0.962 | 1.000 | 1.000 | 0.984 | 0.987 | 0.984 |
| 824 | 0.987 | 0.976 | 0.983 | 0.990 | 0.962 | 1.000 | 1.000 | 0.984 | 0.987 | 0.984 |
| 825 | 0.987 | 0.976 | 0.983 | 0.990 | 0.981 | 1.000 | 1.000 | 0.984 | 0.988 | 0.986 |
| 826 | 0.987 | 0.976 | 0.983 | 0.990 | 0.981 | 1.000 | 1.000 | 0.984 | 0.988 | 0.986 |
| 827 | 0.987 | 0.976 | 0.983 | 0.990 | 0.981 | 1.000 | 1.000 | 0.984 | 0.988 | 0.986 |
| 828 | 0.987 | 0.976 | 0.983 | 0.990 | 0.981 | 1.000 | 1.000 | 0.984 | 0.988 | 0.986 |
| 829 | 0.987 | 0.976 | 0.983 | 0.990 | 0.981 | 1.000 | 1.000 | 0.984 | 0.988 | 0.986 |
| 830 | 0.987 | 0.976 | 0.983 | 0.990 | 0.981 | 1.000 | 1.000 | 0.984 | 0.988 | 0.986 |
| 831 | 0.987 | 0.976 | 0.983 | 0.990 | 0.981 | 1.000 | 1.000 | 0.984 | 0.988 | 0.986 |
| 832 | 0.987 | 0.976 | 0.983 | 0.990 | 0.981 | 1.000 | 1.000 | 0.984 | 0.988 | 0.986 |
| 833 | 0.987 | 0.976 | 0.983 | 0.990 | 0.981 | 1.000 | 1.000 | 0.984 | 0.988 | 0.986 |
| 834 | 0.987 | 0.976 | 0.983 | 0.990 | 0.981 | 1.000 | 1.000 | 0.984 | 0.988 | 0.986 |
| 835 | 0.987 | 0.976 | 0.983 | 0.990 | 0.981 | 1.000 | 1.000 | 0.984 | 0.988 | 0.986 |
| 836 | 0.987 | 0.976 | 0.983 | 0.990 | 0.981 | 1.000 | 1.000 | 0.984 | 0.988 | 0.986 |
| 837 | 0.987 | 0.976 | 0.983 | 0.990 | 0.981 | 1.000 | 1.000 | 0.984 | 0.988 | 0.986 |
| 838 | 0.987 | 0.976 | 0.983 | 0.990 | 0.981 | 1.000 | 1.000 | 0.984 | 0.988 | 0.986 |
| 839 | 0.987 | 0.976 | 0.983 | 0.990 | 0.981 | 1.000 | 1.000 | 0.984 | 0.988 | 0.986 |
| 840 | 0.987 | 0.976 | 0.983 | 0.990 | 0.981 | 1.000 | 1.000 | 0.984 | 0.988 | 0.986 |
| 841 | 0.987 | 0.976 | 0.983 | 0.990 | 0.981 | 1.000 | 1.000 | 0.984 | 0.988 | 0.986 |
| 842 | 0.987 | 0.976 | 0.983 | 0.990 | 0.981 | 1.000 | 1.000 | 0.984 | 0.988 | 0.986 |
| 843 | 0.987 | 0.976 | 0.983 | 0.990 | 0.981 | 1.000 | 1.000 | 0.984 | 0.988 | 0.986 |
| 844 | 0.987 | 0.976 | 0.983 | 0.990 | 0.981 | 1.000 | 1.000 | 0.984 | 0.988 | 0.986 |
| 845 | 0.987 | 0.976 | 0.983 | 0.990 | 0.981 | 1.000 | 1.000 | 0.984 | 0.988 | 0.986 |
| 846 | 0.987 | 0.976 | 0.983 | 0.990 | 0.981 | 1.000 | 1.000 | 0.984 | 0.988 | 0.986 |
| 847 | 0.987 | 0.976 | 0.983 | 0.990 | 0.981 | 1.000 | 1.000 | 0.984 | 0.988 | 0.986 |
| 848 | 0.987 | 0.976 | 0.983 | 0.990 | 0.981 | 1.000 | 1.000 | 0.984 | 0.988 | 0.986 |
| 849 | 0.987 | 0.976 | 0.983 | 0.990 | 0.981 | 1.000 | 1.000 | 0.984 | 0.988 | 0.986 |
| 850 | 0.987 | 0.976 | 0.983 | 0.990 | 0.981 | 1.000 | 1.000 | 0.984 | 0.988 | 0.986 |
| 851 | 0.987 | 0.976 | 0.983 | 0.990 | 0.981 | 1.000 | 1.000 | 0.984 | 0.988 | 0.986 |
| 852 | 0.987 | 0.976 | 0.983 | 0.990 | 0.981 | 1.000 | 1.000 | 0.984 | 0.988 | 0.986 |
| 853 | 0.987 | 0.976 | 0.983 | 0.990 | 0.981 | 1.000 | 1.000 | 0.984 | 0.988 | 0.986 |
| 854 | 0.987 | 0.976 | 0.983 | 0.990 | 0.981 | 1.000 | 1.000 | 0.984 | 0.988 | 0.986 |
| 855 | 0.987 | 0.976 | 0.983 | 0.990 | 0.981 | 1.000 | 1.000 | 0.984 | 0.988 | 0.986 |
| 856 | 0.987 | 0.976 | 0.983 | 0.990 | 0.981 | 1.000 | 1.000 | 0.984 | 0.988 | 0.986 |
| 857 | 0.987 | 0.976 | 0.983 | 0.990 | 0.981 | 1.000 | 1.000 | 0.984 | 0.988 | 0.986 |
| 858 | 0.987 | 0.976 | 0.983 | 0.990 | 0.981 | 1.000 | 1.000 | 0.984 | 0.988 | 0.986 |
| 859 | 0.987 | 0.976 | 0.983 | 0.990 | 0.981 | 1.000 | 1.000 | 0.984 | 0.988 | 0.986 |
| 860 | 0.987 | 0.976 | 0.983 | 0.990 | 0.981 | 1.000 | 1.000 | 0.984 | 0.988 | 0.986 |
| 861 | 0.987 | 0.976 | 0.983 | 0.990 | 0.981 | 1.000 | 1.000 | 0.984 | 0.988 | 0.986 |
| 862 | 0.987 | 0.976 | 0.983 | 0.990 | 0.981 | 1.000 | 1.000 | 0.984 | 0.988 | 0.986 |
| 863 | 0.987 | 0.976 | 0.983 | 0.990 | 0.981 | 1.000 | 1.000 | 0.984 | 0.988 | 0.986 |
| 864 | 0.987 | 0.976 | 0.983 | 0.990 | 0.981 | 1.000 | 1.000 | 0.984 | 0.988 | 0.986 |
| 865 | 0.987 | 0.976 | 0.983 | 0.990 | 0.981 | 1.000 | 1.000 | 0.984 | 0.988 | 0.986 |
| 866 | 0.987 | 0.976 | 0.983 | 0.990 | 0.981 | 1.000 | 1.000 | 0.984 | 0.988 | 0.986 |
| 867 | 0.987 | 0.976 | 0.983 | 0.990 | 0.981 | 1.000 | 1.000 | 0.984 | 0.988 | 0.986 |
| 868 | 0.987 | 0.976 | 0.983 | 0.990 | 0.981 | 1.000 | 1.000 | 0.984 | 0.988 | 0.986 |
| 869 | 0.987 | 0.976 | 0.983 | 0.990 | 0.981 | 1.000 | 1.000 | 0.984 | 0.988 | 0.986 |
| 870 | 0.987 | 0.976 | 0.983 | 0.990 | 0.981 | 1.000 | 1.000 | 0.984 | 0.988 | 0.986 |
| 871 | 0.987 | 0.976 | 0.983 | 0.990 | 0.981 | 1.000 | 1.000 | 0.984 | 0.988 | 0.986 |
| 872 | 0.987 | 0.976 | 0.983 | 0.990 | 0.981 | 1.000 | 1.000 | 0.984 | 0.988 | 0.986 |
| 873 | 0.987 | 0.976 | 0.983 | 0.990 | 0.981 | 1.000 | 1.000 | 0.984 | 0.988 | 0.986 |
| 874 | 0.987 | 0.976 | 0.983 | 0.990 | 0.981 | 1.000 | 1.000 | 0.984 | 0.988 | 0.986 |
| 875 | 0.987 | 0.976 | 0.983 | 0.990 | 0.981 | 1.000 | 1.000 | 0.984 | 0.988 | 0.986 |
| 876 | 0.987 | 0.976 | 0.983 | 0.990 | 0.981 | 1.000 | 1.000 | 0.984 | 0.988 | 0.986 |
| 877 | 0.987 | 0.976 | 0.983 | 0.990 | 0.981 | 1.000 | 1.000 | 0.984 | 0.988 | 0.986 |
| 878 | 0.987 | 0.976 | 0.983 | 0.990 | 0.981 | 1.000 | 1.000 | 0.984 | 0.988 | 0.986 |
| 879 | 0.987 | 0.976 | 0.983 | 0.990 | 0.981 | 1.000 | 1.000 | 0.984 | 0.988 | 0.986 |
| 880 | 0.987 | 0.976 | 0.983 | 0.990 | 0.981 | 1.000 | 1.000 | 0.984 | 0.988 | 0.986 |
| 881 | 0.987 | 0.976 | 0.983 | 0.990 | 0.981 | 1.000 | 1.000 | 0.984 | 0.988 | 0.986 |
| 882 | 0.987 | 0.976 | 0.983 | 0.990 | 0.981 | 1.000 | 1.000 | 0.984 | 0.988 | 0.986 |
| 883 | 0.987 | 0.976 | 0.983 | 0.990 | 0.981 | 1.000 | 1.000 | 0.984 | 0.988 | 0.986 |
| 884 | 0.987 | 0.976 | 0.983 | 0.990 | 0.981 | 1.000 | 1.000 | 0.984 | 0.988 | 0.986 |
| 885 | 0.987 | 0.976 | 0.983 | 0.990 | 0.981 | 1.000 | 1.000 | 0.984 | 0.988 | 0.986 |
| 886 | 0.987 | 0.976 | 0.983 | 0.990 | 0.981 | 1.000 | 1.000 | 0.984 | 0.988 | 0.986 |
| 887 | 0.987 | 0.976 | 0.983 | 0.990 | 0.981 | 1.000 | 1.000 | 0.984 | 0.988 | 0.986 |
| 888 | 0.987 | 0.976 | 0.983 | 0.990 | 0.981 | 1.000 | 1.000 | 0.984 | 0.988 | 0.986 |
| 889 | 0.987 | 0.976 | 0.983 | 0.990 | 0.981 | 1.000 | 1.000 | 0.984 | 0.988 | 0.986 |
| 890 | 0.987 | 0.976 | 0.983 | 0.990 | 0.981 | 1.000 | 1.000 | 0.984 | 0.988 | 0.986 |
| 891 | 0.987 | 0.976 | 0.983 | 0.990 | 0.981 | 1.000 | 1.000 | 0.984 | 0.988 | 0.986 |
| 892 | 0.987 | 0.976 | 0.983 | 0.990 | 0.981 | 1.000 | 1.000 | 0.984 | 0.988 | 0.986 |
| 893 | 0.987 | 0.976 | 0.983 | 0.990 | 0.981 | 1.000 | 1.000 | 0.984 | 0.988 | 0.986 |
| 894 | 0.987 | 0.976 | 0.983 | 0.990 | 0.981 | 1.000 | 1.000 | 0.984 | 0.988 | 0.986 |
| 895 | 0.987 | 0.976 | 0.983 | 0.990 | 0.981 | 1.000 | 1.000 | 0.984 | 0.988 | 0.986 |
| 896 | 0.987 | 0.976 | 0.983 | 0.990 | 0.981 | 1.000 | 1.000 | 0.984 | 0.988 | 0.986 |
| 897 | 0.987 | 0.976 | 0.983 | 0.990 | 0.981 | 1.000 | 1.000 | 0.984 | 0.988 | 0.986 |
| 898 | 0.987 | 0.976 | 0.983 | 0.990 | 0.981 | 1.000 | 1.000 | 0.984 | 0.988 | 0.986 |
| 899 | 0.987 | 0.976 | 0.983 | 0.990 | 0.981 | 1.000 | 1.000 | 0.984 | 0.988 | 0.986 |
| 900 | 0.987 | 0.976 | 0.983 | 0.990 | 0.981 | 1.000 | 1.000 | 0.984 | 0.988 | 0.986 |
